# Alcohol use disorder and disability insurance in Switzerland: the attitudes and views of lawyers, insurance medical experts, and addiction-specialist therapists

**DOI:** 10.1186/s13011-022-00495-x

**Published:** 2022-10-27

**Authors:** Helen Wyler, Anja Maisch, Thomas Berger, Ueli Kieser, Roman Schleifer, Michael Liebrenz

**Affiliations:** 1 Department of Forensic Psychiatry, University of Bern, Falkenplatz 16/18, 3012 Bern, Switzerland; 2 Department of Clinical Psychology and Psychotherapy, University of Bern, Bern, Switzerland; 3 Institute for Legal Studies and Legal Practice, University of St. Gallen, St. Gallen, Switzerland

**Keywords:** Alcohol use disorder, Disability insurance, Lawyers, Insurance medical experts, Addiction-specialist therapists, Disease model of addiction, Moralist/choice view, Switzerland

## Abstract

**Background:**

According to a landmark decision by the Swiss Federal Supreme Court, people with a substance use disorder (SUD) are now eligible for disability benefits if their disorder impairs their ability to work. Alcohol use disorder (AUD) is one of the most common SUDs in Switzerland and is associated with high societal and economic costs. This study aimed to gain an in-depth understanding of the views of professional stakeholder groups regarding AUD and their opinions on the new legal precedent.

**Methods:**

Swiss social insurance lawyers, insurance medical experts, and addiction-specialist therapists (*N* = 79) answered an online questionnaire. Due to violations of the assumption of normality, non-parametric tests are reported in most cases.

**Results:**

Therapists held significantly higher regard for patients with AUD than both lawyers and insurance medical experts. All three groups strongly supported a disease view of AUD but agreed significantly less that it was a disease like cancer, suggesting that AUDs might be seen as at least partially self-inflicted. Overall, moralist views of AUD received considerably less support than the disease view, with lawyers agreeing with moralist views more than therapists. All groups were well-informed and largely supportive about the new legal precedent. When asked about stipulating participation in medical treatment to mitigate damages associated with a claim, attending therapy was supported the most amongst the groups (80% of participants felt this was somewhat or fully appropriate), followed by a reduction in drinking quantity (58%), and abstinence (18%). In all three groups, we identified associations between certain views and opinions on AUD and support for the new legal precedent.

**Conclusions:**

Whilst there were differences between the stakeholder groups in their regard for and views of AUD, all three adopted a clear harm-reduction approach with respect to measures to mitigate damages associated with the insurance disability claim. A possible connection of this stance with the Swiss national drug policy in recent years is discussed together with limitations of the study and practical implications of the findings.

**Supplementary Information:**

The online version contains supplementary material available at 10.1186/s13011-022-00495-x.

## Background

Worldwide, alcohol consumption and alcohol use disorders (AUDs) have high social and economic costs [1, 2]. Switzerland is no exception [3], with AUD being one of the most prevalent substance use disorders (SUDs) [4]. Significantly, AUD also is an important risk factor for disability-adjusted life years in young people, with specific links to work disability [5]. Concurrently, AUD is a highly stigmatised condition, more so than other mental illnesses [6, 7], and even amongst health professionals [8]. Such negative conceptions can influence quality of care and can lead to worse treatment outcomes [9], treatment dropout [10], or avoidant treatment approaches [11]. The area of disability insurance encapsulates these different aspects. The wider process required to assess the occupational capacity of a person with AUD involves professionals from different fields, whose individual views and attitudes can potentially influence said outcome.

### Swiss disability insurance and recent changes in legislation

In Switzerland, social security in cases of disability or chronic illness is mainly covered by disability insurance [12]. Whilst the most recent revision to disability insurance legislation was introduced in 2022, the legal practice had seen numerous changes since the previous revision in 2012. These changes were initiated by rulings of the Swiss Federal Supreme Court (legal precedents) and are only officially binding for the lower court whose case the Supreme Court considered. However, lower courts do not typically diverge from legal precedents to avoid the Supreme Court overturning their judgement if brought before it.

Following a landmark decision by the Swiss Federal Supreme Court in 2015, the implications of a psychiatric disorder on the ability to work must be assessed through a structured evidentiary procedure (*strukturiertes Beweisverfahren*) (BGE 141 V 281; BGE 143 V 418) [13]. Importantly, however, this ruling did not establish equivalence between SUDs and other psychiatric disorders, such as schizophrenia or depression. The Court continued to insist that the direct effects of SUDs were not applicable under disability insurance law; for the Court, SUDs were “surmountable by an effort of will” (*durch Willensanstrengung überwindbar*) and thus not a medical illness [13].

In 2019, the Swiss Federal Supreme Court finally amended this position. Based on a thorough review of current scientific knowledge, the Court ruled that SUDs are an illness and must be treated like any other psychiatric disorder (BGE 145 V 215/verdict 9C_724/2018; for a discussion, see e.g. [14]). Amongst others, the Court cited two publications written by three of the authors of the present paper [15, 16] published in a local journal on social insurance law. This underlines the value of making relevant scientific knowledge accessible to a legal audience to effect change. Of course, akin to other psychiatric disorders, people with an SUD have an obligation to mitigate damages, for example, by participating in reasonable medical treatment (BGE 145 V 215/verdict 9C_724/2018).

This legal precedent reflects a paradigm shift from a morally-shaped model of addiction to a disease model of addiction. In a moral model, drug use is deemed a choice, and a critical moral stance is adopted against this choice ([17], p. 170). Consequently, people with an addiction should be held responsible for this self-inflicted condition. Contrastingly, disease models of addiction, such as the brain disease model of addiction (for a critical discussion of this specific model, see e.g. [18]), assume that addictive behaviour is caused by disease symptomatology and cannot be helped ([19], p. 117, [20]). Thus, addiction is perceived as a (medical) illness.

Prior to this Swiss jurisprudential development, there was a long-standing inconsistency between legislation and the prevailing medical views about SUDs. This discrepancy is noteworthy since closer adherence to a moral model and the perception that people with AUD are responsible for their condition has been associated with increased stigmatisation [21, 22] and negative resource allocation decisions by the public [23]. The picture relating to a medical, neurobiological disease, or brain disease model of addiction is less clear, with some studies finding reduced stigma [21, 24] and others reporting no association or negative effects [22, 25]. In sum, endorsing a moral model of addiction in particular seems to adversely affect levels of stigmatisation. An important question, therefore, is to what extent ideas related to the moral model still prevail amongst relevant stakeholders and whether they are associated with stakeholders’ support of the new legal practice.

### Attitudes and stigma amongst relevant professional groups

We identified four professional occupational groups involved in the assessment of an SUD disability insurance case: general practitioners (GPs) as a likely first point of contact and specialist treatment gatekeepers; insurance medical experts, who assess ability to work; social security lawyers, who provide social security and pension advice and represent clients in court; and addiction-specialist therapists, who can be involved at any stage of a case. To our knowledge, there is scant research about AUD perceptions amongst these professional groups in Switzerland. Moreover, scarce evidence exists about insurance medical experts and social insurance lawyers in international literature.

Various studies have examined the attitudes of healthcare professionals, including GPs and addiction-specialist therapists, towards people with AUD in the US, the UK, and EU countries [26]. A systematic review of largely US-based studies examining drug and alcohol treatment providers’ views found support for a disease model amongst respondents, such as addiction physicians, psychologists, and social service staff [26]. Yet, some of these papers also noted that respondents favoured alternative models, and, interestingly, that supporting a disease model of addiction does not preclude support for other seemingly conflicting models ([26], p. 715), such as the free-will model (e.g. [27]) or a moral model (e.g. [28]). Similarly, Rosta [29] observed that doctors in Denmark and Germany viewed addiction as a disease, but almost half also indicated that the illness was self-induced. Another study found that GPs and the public endorsed the idea that addiction was due to weakness slightly more than mental health and addiction specialists ([30], p. 5), even though all professional groups agreed to a similar extent that addiction was a disease; the public supported this view less. They also reported that GPs and the public endorsed slightly more negative stereotypical beliefs than specialists. Thus, stereotypical beliefs and conceptions in line with a non-disease model of addiction can also be observed in groups such as GPs [30] and addiction-specialist therapists [28].

Avery et al. [31] investigated attitudes towards the brain disease model of addiction for SUDs in US physicians and (criminal defence) attorneys. In both groups, supporting this model was associated with more positive attitudes compared to considering addiction as a failure in self-control or a moral lapse. It should be noted, however, that the latter two conceptions were rarely endorsed (totals of 3.9% and 6.3% for attorneys and physicians, respectively). Conversely, only 10% of surveyed attorneys in a Nigerian sample considered SUDs to be a medical (psychiatric) concern [32].

We are not aware of studies examining insurance medical experts’ perceptions about SUD or AUD. Although they are physicians with additional training to provide specialist opinions, it is possible that insurance medical experts’ views differ from other health professionals. This may be because insurance medicine might attract people with specific attitudes and opinions, or because of the specific frameworks in which assessments are made. Literature on role conflicts in medicine suggests that payment by insurance companies in order to provide disability assessments may even lead to potential allegiance to the payer [33, 34].

### The present study

This study aimed to examine the attitudes of professional stakeholder groups (GPs, addiction-specialist therapists, insurance medical experts, and social insurance lawyers) concerning AUD and the new Swiss legal precedent. Specifically, we were interested in professionals’ (1) regard for AUD, (2) consideration of AUD as a disease, (3) support for a moralist view of AUD, (4) perception of the on- and offset responsibility for AUD, and (5) treatment beliefs, particularly regarding abstinence. Moreover, we aimed to investigate stakeholders’ opinions on the new legal precedent and on different medical treatments as part of the insured person’s duty to mitigate damages. Finally, we intended to explore if views more aligned with the former legal practice were related to respondents’ level of support for the new legal precedent. The results can inform education and training requirements; previous research has shown that beliefs about AUD are modifiable, and experience and education can improve negative attitudes [35, 36].

## Method

The findings presented here form part of a larger project funded by the Swiss Foundation for Alcohol Research (SSA 305) on stakeholders’ perceptions of AUD and the new legal precedent. The quantitative findings regarding stakeholders’ views on AUD and the new legal precedent are presented below. Further results on case vignettes and from qualitative focus groups on stakeholders’ first experiences with the new legislation are beyond the scope of this paper.

### Study design

The final design was one-factorial with 3 groups (lawyers, insurance medical experts, and addiction-specialist therapists). The group of general practitioners who did not also work as addiction-specialist therapists or insurance medical experts was too small to be included in the analyses (see Participants).

### Measures

All materials were prepared in German and then translated to French and Italian by two bilingual members of our forensic-psychiatric service.

#### Medical Condition Regard Scale

The Medical Condition Regard Scale (MCRS) is an 11-item scale with good construct validity and reliability to assess professionals’ regard towards different medical conditions [37, 38]. We specifically adapted the MCRS for AUD and translated it into German. Responses were assessed on a 6-point scale, ranging from 1 strongly disagree to 6 strongly agree. We calculated an overall sum score, with higher scores indicating higher regard for AUD. The MCRS scale was only presented to participants who indicated having at least some work-related contact with individuals with AUD (n = 78 in the final sample). Missing values were replaced with the mean of the other items for up to two missing answers (n = 1) [30]. Reponses from four participants were excluded from the analyses as more than two answers were missing. Cronbach’s Alpha of the scale was good (0.87).

#### Views and opinions on AUD

To assess participants’ views and opinions on AUD, we created a questionnaire based on items that were adapted from various sources, with some items being self-developed by two of the study’s authors with extensive experience in the field of SUDs (see Table 1 for an overview). The original German wording is included in the Supplemental Online Materials.

**Table 1:** Items on Views of and Opinions on AUD (Including their Source)

No	Item	Source
	**Disease view**	
1	Alcohol dependence is a disease	adapted from [30] and [39]
2	Alcohol dependence is a disease like cancer: you cannot help having it	self-developed
3	Alcohol dependence is best understood as a disease of the brain	adapted from [40]
4	Alcohol dependence is a disease of the psyche	adapted from [40]
	**Moral view**	
5	Alcohol dependence is an expression of weakness of character	adapted from [40]
6	Alcohol dependence is an expression of weakness of will	adapted from [30] and [40]
7	A person with alcohol dependence lacks self-discipline	adapted from [24]
	**On- and offset responsibility**	
8	A person with alcohol dependence is responsible for the development of his/her addiction	adapted from [39]
9	A person with alcohol dependence is responsible for managing their own addiction	adapted from [39]
	**Treatment-related beliefs**	
10	A person with alcohol dependence can be treated successfully	adapted from [30]
11	In principle, withdrawal is reasonable for a person with alcohol dependence	self-developed
12	The goal of an intervention for alcohol dependence should always be abstinence	adapted from [39]
13	When a person with alcohol dependence goes through withdrawal, this leads to a permanent improvement of their ability to work	self-developed

The items assessed participants’ views on the disease model of addiction (items 1–4 in Table 1), the moral model (items 5–7), onset responsibility (considering a person to be responsible for developing AUD; item 8), offset responsibility (considering a person to be responsible for recovery; item 9), and some treatment-related views, with a particular focus on the importance participants ascribed to withdrawal treatment and abstinence (items 10–13). All items were assessed on a five-point Likert scale, ranging from do not agree at all (1) to fully agree (5).

#### Opinions and knowledge about the new legal precedent

Given our investigation’s aims, we used self-developed questions to explore participants’ knowledge and opinions about the new Swiss legal precedent and potential mitigating measures. We first asked participants whether they were aware of the new legal precedent (yes/no). After providing information on the new legal precedent, we asked participants if, in principle, they felt it was appropriate (“richtig”) that a person who is not or only partially able to work due to alcohol dependence can receive a disability insurance pension (definitely no, somewhat no, neither no nor yes, somewhat yes, definitely yes). Furthermore, we were interested in how suitable participants felt three therapeutic measures were (complete abstinence, reduction in drinking quantity, and attending therapy) that could be imposed to mitigate damages associated with the claim (in Switzerland, not observing a measure can result in sanctions such as pension reductions). Responses could range from not at all appropriate (1) to fully appropriate (5) on a five-point Likert scale.

#### Demographics

We assessed age, gender, nationality, years of work experience, urbanisation of the place of practice, language usually spoken with clients, and frequency of contact with persons with AUD (see Table 2 for response categories). Further, we asked what description(s) best fit participants’ work (doctor at the disability insurance (excluding external consultants), external consultant for the disability insurance, therapist in the field of SUDs, therapist in the general psychiatric-psychotherapeutic field, lawyer in social insurance law, lawyer in another field (please specify), other (please specify); multiple responses were possible).

**Table 2:** Descriptive Information on the Three Stakeholder Groups

	Lawyers % (*N*)	Insurance medical experts % (*N*)	Addiction-specialist therapists % (*N*)	*p*
	*N* = 28	*N* = 21	*N* = 30	
Mean age (± SD)	51.75 (9.22)	60.38 (9.92)	50.10 (9.01)	< .001
Gender				.841
Men	60.7 (17)	66.7 (14)	56.7 (17)	
Women	39.3 (11)	33.3 (7)	40.0 (12)*	
Nationality				.109
Swiss (incl. dual citizens)	78.6 (22)	57.1 (12)	53.3 (16)	
Other	21.4 (6)	42.9 (9)	46.7 (14)	
Urbanisation in place of practice				.077
Urban	67.9 (19)	42.9 (9)	60.0 (18)	
Agglomeration	32.1 (9)	33.3 (7)	26.7 (8)	
Rural	0.0 (0)	23.8 (5)	13.3 (4)	
Main language with clients^1^				
German	82.1 (23)^a^	71.4 (15)^a,b^	50.0 (15)^b^	.034
French	17.9 (5)^a^	19.0 (4)^a^	53.3 (16)^b^	.005
Italian	3.6 (1)	14.3 (3)	13.3 (4)	.404
Frequency of working with AUD clients	^a^	^b^	^c^	< .001
Daily	0.0 (0)	0.0 (0)	56.7 (17)	
Weekly	3.6 (1)	28.6 (6)	36.7 (11)	
Monthly	10.7 (3)	28.6 (6)	3.3 (1)	
A few times a year	42.9 (12)	38.1 (8)	3.3 (1)	
Once a year or less	39.3 (11)	4.8 (1)	0.0 (0)	
Never	3.6 (1)	0.0 (0)	0.0 (0)	

^*^ One person did not specify their gender. ^1^ Multiple responses per participant possible

### Procedure

The survey was conducted using an online questionnaire presented with Qualtrics®. Participants received a personally addressed email with a short project description and an anonymous survey link. After providing consent by ticking the relevant boxes and answering demographic questions, participants were presented with the questionnaires. Participants did not receive payment. The University’s Phil.-Hum. Faculty Ethics Committee approved this study.

The questionnaires were available in three of the four official Swiss languages (German, French, and Italian), which allowed for nationwide recruitment (the fourth official language, Romansh, is spoken only by 0.5% of the population; [41]). Using occupation-specific online member directories, 100 professionals per group (i.e. a total of 400) were randomly selected and contacted via email to partake in our online survey. If no email address was available online, the person/practice was contacted via telephone to obtain an email address. If a person could not be contacted, he or she was omitted and the next person on the directory was contacted. The selected professionals were contacted three times (first contact, first reminder, second reminder) via email between January and June 2021. Emails were personally addressed. In an attempt to increase the response rate, reminders were group-specific and highlighted why we were particularly interested in the views of each professional group.

### Participants

Of the 400 people we contacted, 112 participated in the survey (28%). After eliminating those who did not finish the main part of the survey (*n* = 15) or who left too many questions unanswered (*n* = 1), the sample consisted of 96 participants (overall response rate of 24%). Response rate was 23% for medical professionals and 28% for lawyers. To group health professionals, who could fit into more than one category (e.g. working as a GP and as an insurance medical expert), we proceeded as follows: any participant who reported working as a therapist specialised in the field of SUDs was allocated to the *addiction specialist therapist* group (*n* = 30). Of those remaining, participants who reported working as a medical expert were allocated to the *insurance medical expert* group (*n* = 21)*.* For the remaining participants, nine reported working as GPs and eight identified as general therapists without addiction-specialism; although participants were contacted via professional registers, it is possible that some have not yet started to work or ceased working in that particular field. As the two groups were highly heterogeneous, we were concerned about the meaningfulness of any results reported for collapsed data, which is why we did not include data from these participants in our analyses. Finally, any participant who reported working as a lawyer was allocated to the *legal experts* group (*n* = 28). Thus, the final sample consisted of 79 participants.

Descriptive information on the three professional groups can be seen in Table 2. As expected, addiction-specialist therapists worked significantly more frequently with people with AUD than insurance medical experts (*p*_B_ < 0.001, *r* = 0.53), who, in turn, had more contact with people with AUD than lawyers (*p*_B_ = 0.030, *r* = 0.37). The higher age in insurance medical experts compared to the other two groups is likely because working as an insurance medical expert requires additional training, and some physicians continue working as insurance medical experts beyond retirement age.

### Statistical analyses

All analyses were conducted with SPSS 28. An alpha level of 0.05 was set and all tests are reported two-tailed. Where assumptions were violated, we report Kruskal–Wallis tests, Wilcoxon Signed Rank tests, and Friedman tests together with the Bonferroni-Holm corrected *p*-values (*p*_*BHc*_). Follow-up pairwise comparisons are reported with the normal Bonferroni correction for multiple tests (*p*_B_). The effect size *r* is reported for the follow-up pairwise comparisons and the Wilcoxon Signed Rank tests [42].

## Results

### MCRS

There was a statistically significant difference in the MCRS score between the three groups, *F*(2, 72) = 32.00, *p* < 0.001, *ω*^*2*^ = 0.45. Addiction-specialist therapists (*M* = 56.63, *SD* = 6.09) showed significantly higher regard than both lawyers (*M* = 44.25, *SD* = 6.86; *p* < 0.001) and insurance medical experts (*M* = 44.24, *SD* = 6.89; *p* < 0.001), with the difference between the latter two being non-significant (*p* = 1.000). There was no statistically significant correlation between frequency of working with AUD clients and the MCRS scores in the individual groups, all *p*_BHC_ > 0.164.

### Views and beliefs about AUD

Table 3 provides an overview on the mean, standard deviation, and median per group for the individual items assessing views of and beliefs about AUD.

**Table 3:** Mean, Standard Deviation, and Median for Views and Beliefs About AUD in the Three Groups

	Lawyers		Insurance medical experts		Addiction-specialist therapists		*p*_*BHc*_
	M (SD)	Mdn	M (SD)	Mdn	M (SD)	Mdn	
**Disease view**							
Alcohol dependence is a disease	4.79 (0.42)	5.0	4.71 (0.46)	5.0	4.93 (0.25)	5.0	.110
Alcohol dependence is a disease like cancer: you cannot help having it	2.82 (1.19)	3.0	3.13 (1.11)	3.0	3.67 (1.30)	4.0	.066
Alcohol dependence is best understood as a disease of the brain	2.93 (1.09)	3.0	3.30 (1.13)	3.5	3.67 (1.30)	4.0	.104
Alcohol dependence is a disease of the psyche	3.96 (0.79)	4.0	3.95 (0.74)	4.0	4.47 (0.63)	5.0	.052
**Moralist/choice view**							
Alcohol dependence is an expression of weakness of character	1.86 (1.01)	2.0	1.43 (0.60)	1.0	1.20 (0.61)	1.0	.008
Alcohol dependence is an expression of weakness of will	2.18 (1.16)	2.0	1.62 (1.02)	1.0	1.30 (0.84)	1.0	.003
A person with alcohol dependence lacks self-discipline	2.32 (1.06)	2.0	1.86 (1.01)	2.0	1.67 (1.03)	1.0	.027
**On- and offset responsibility**							
A person with alcohol dependence is responsible for the development of his/her addiction	2.46 (1.11)	2.5	2.20 (1.06)	2.0	1.87 (0.94)	2.0	.208
A person with alcohol dependence is responsible for managing their own addiction	2.29 (1.30)	2.0	2.14 (1.11)	2.0	2.60 (1.38)	3.0	.480
**Treatment-related views**							
A person with alcohol dependence can be treated successfully	4.07 (0.73)	4.0	4.24 (0.83)	4.0	4.53 (0.73)	5.0	.081
In principle, withdrawal is reasonable for a person with alcohol dependence	3.96 (0.71)	4.0	3.86 (0.66)	4.0	3.87 (0.82)	4.0	.866
The goal of an intervention for alcohol dependence should always be abstinence	2.93 (1.12)	3.0	2.76 (1.45)	3.0	1.90 (1.19)	1.5	.036
When a person with alcohol dependence goes through withdrawal, this leads to a permanent improvement of their ability to work	3.36 (1.10)	4.0	3.05 (0.92)	3.0	2.73 (1.02)	3.0	.082

#### Disease view of AUD

The three groups strongly agreed with the statement that AUD was a disease (see Table 3). No statistically significant between-group differences were observed, *H*(2) = 4.42, *p*_*BHc*_ = 0.110. In comparison, all three groups agreed less with the statement that AUD was a disease like cancer in the sense that the affected person was not at fault (all *p*_*BHc*_ < 0.001, all *r* > 0.49). The groups also differed in the extent of their agreement with this statement, *H*(2) = 7.61, *p*_*BHc*_ = 0.066; agreement was higher amongst addiction-specialist therapists than lawyers (*p*_*B*_ = 0.020, *r* = 0.36). Further, post-hoc exploratory analyses showed a moderate negative correlation between the statement that AUD was a disease like cancer and perceived on-set responsibility, *r*_s_ = -0.58, *p* < 0.001. Thus, the element of perceived self-infliction may be of relevance for those who agreed less that AUD was a disease like cancer.

There was no statistically significant difference between the three groups in their support of the brain disease model, *H*(2) = 5.93, *p*_*BHc*_ = 0.104. By contrast, addiction-specialist therapists tended to agree more strongly with the statement that AUD was a disease of the psyche than lawyers (*p*_*B*_ = 0.031, *r* = 0.34) and insurance medical experts (*p*_*B*_ = 0.041, *r* = 0.35), *H*(2) = 8.76, *p*_*BHc*_ = 0.052. Support for AUD as a disease of the psyche was statistically significantly higher than support for the brain disease model in all three groups (all *p*_*BHc*_ < 0.026, all *r* > 0.34).

#### Moralist/choice view of AUD

Kruskal–Wallis tests yielded statistically significant differences between the groups regarding the statement that AUD was as a consequence of a weak character, *H*(2) = 10.92, *p*_*BHc*_ = 0.008, a weak will, *H*(2) = 13.42, *p*_*BHc*_ = 0.003, and a lack of self-discipline, *H*(2) = 7.25, *p*_*BHc*_ = 0.027. For all three items, lawyers agreed with the statements statistically significantly more than addiction-specialist therapists (*p*_*B*_ = 0.003, *r* = 0.43, *p*_*B*_ = 0.001, *r* = 0.48, and *p*_*B*_ = 0.023, *r* = 0.35, respectively), with insurance medical experts scoring between the two groups. Overall, most respondents across all groups fully or partially disagreed with each of these statements, as reflected in the low mean and median scores (see Table 3).

#### Perceived responsibility

No statistically significant differences were observed regarding perceived on- and offset responsibility between the groups, *H* (2) = 4.53, *p*_*BHc*_ = 0.208, and *H* (2) = 1.47, *p*_*BHc*_ = 0.480, respectively.

#### Treatment beliefs

The groups tended to diverge in the extent to which they felt that AUD could be treated successfully, *H* (2) = 7.20, *p*_*BHc*_ = 0.081, with addiction-specialist therapists being more positive than lawyers (*p*_*B*_ = 0.023, *r* = 0.35). There was no statistically significant difference in the three groups’ agreement that withdrawal treatment was reasonable in principle for a person with AUD, *H* (2) = 0.29, *p*_*BHc*_ = 0.866. However, they tended to differ in supporting the view that undergoing withdrawal would result in a permanent improvement of a person’s ability to work, *H* (2) = 6.38, *p*_*BHc*_ = 0.082, with lawyers being more optimistic than addiction-specialist therapists (*p*_*B*_ = 0.036, *r* = 0.33). Finally, addiction-specialist therapists agreed significantly less that the intervention goal for AUD should always be abstinence compared with lawyers (*p*_B_ = 0.004, *r* = 0.42) and, by trend, insurance medical experts (*p*_B_ = 0.063, *r* = 0.32), *H* (2) = 11.16, *p*_*BHc*_ = 0.036. Interestingly, whilst 77% of addiction-specialist therapists disagreed completely or partially with the latter statement and only 10% agreed completely or partially, a bi-modal distribution was observed for both insurance medical experts (47% completely or partially disagreed, 43% completely or partially agreed) and lawyers (39% completely or partially disagreed, 39% completely or partially agreed).

### The new legal precedent: knowledge and attitudes

The three groups did not statistically significantly differ in the extent to which they reported being aware of the new legal precedent (lawyers: 89%; insurance medical experts: 95%; addiction-specialist therapists: 80%), Fisher’s exact test = 2.45, *p* = 0.293. Most participants answered “yes” (68%) or “somewhat yes” (24%) to the question whether they personally considered it to be right in principle that a person with AUD can receive disability benefit pension as per the new legal precedent; there were no statistically significant differences between the groups, *H* (2) = 2.59, *p* = 0.275.

Across all groups, 18% of participants felt that requesting abstinence was somewhat or fully appropriate as a potential damage mitigation measure, as compared to 58% for a reduction in drinking quantity and 80% for therapy. In all three stakeholder groups, there were statistically significant differences in the extent to which they supported these various measures (Friedman’s tests; all *p*_*BHc*_ < 0.001; see Table 4 for mean and median values per group and details on group differences). Whilst lawyers and insurance medical experts felt requesting abstinence was less appropriate than requesting a reduction in drinking quantity or attending therapy, addiction-specialist therapists additionally felt that reduced drinking was less appropriate than attending therapy (see Table 4). Addiction-specialist therapists had split views on the appropriateness of reduced drinking, with 47% feeling it was not at all or somewhat not appropriate vs. 37% indicating it was somewhat or fully appropriate.

**Table 4:** Perceived Appropriateness of Imposing Different Therapeutic Measures to Mitigate Damages

	Lawyers		Insurance medical experts		Addiction-specialist therapists	
	*M* (*SD*)	Mdn	*M* (*SD*)	Mdn	*M* (*SD*)	Mdn
Abstinence	2.38 (1.24)	2.0^a^	2.57 (1.33)	2.0^a^	1.77 (1.07)	1.0^a^
Reduction in drinking quantity	3.62 (1.44)	4.0^b^	3.67 (1.16)	4.0^b^	2.63 (1.40)	3.0^b^
Therapy	4.00 (1.41)	4.5^b^	3.90 (1.22)	4.0^b^	3.64 (1.07)	4.0^c^

Different letters identify statistically significant differences between two measures *within* a group

By trend, the groups differed in their support for imposing abstinence as a measure to mitigate damages, *H* (2) = 7.19, *p*_*BHc*_ = 0.054, with addiction-specialist therapists being more opposed to that request than insurance medical experts (*p*_B_ = 0.043, *r* = 0.34) and, at the descriptive level, also more so than lawyers (*p*_B_ = 0.121, *r* = 0.27) (see Table 4). The groups statistically significantly differed in their support for imposing a reduction in drinking quantity, *H* (2) = 9.94, *p*_*BHc*_ = 0.021, with addiction-specialist therapists being more opposed toward this measure than insurance medical experts (*p*_B_ = 0.040, *r* = 0.35) and lawyers (*p*_B_ = 0.014, *r* = 0.37). There were no statistically significant differences between the groups in their support of the request to attend therapy, *H* (2) = 0.67, *p*_*BHc*_ = 0.717.

### Relationship between attitudes towards the new legal precedent and views of AUD

Exploratory analyses of the associations between agreeing that it was right in principle that people with AUD could get disability pension benefits and several items related to the old vs. new legal practice were conducted at a group level: AUD is a disease / a disease like cancer (positive correlations expected with support of new legal precedent); AUD is due to a weak will / weak character / lack of self-discipline (negative correlations expected); perception that an individual is responsible for the on- and off-set of their condition (negative correlations expected). Full correlation tables per group are available in the Supplemental Materials.

For lawyers (*N* = 28), Spearman correlations showed that those who agreed more with the new legal precedent also agreed more that AUD was a disease (*r*_*s*_ = 0.44, *p* = 0.018) and that AUD was a disease like cancer (*r*_*s*_ = 0.40, *p* = 0.035). Moreover, they agreed less that AUD was due to weak will (*r*_*s*_ = -0.75,* p* < 0.001), weak character (*r*_*s*_ = -0.45, *p*_*BHc*_ = 0.016), and lack of self-discipline (*r*_*s*_ = -0.62,* p* < 0.001), and also ascribed lower on-set responsibility (*r*_*s*_ = -0.42,* p* = 0.028). No statistically significant correlation was observed for off-set responsibility (*r*_*s*_ = -0.04, *p* = 0.832).

For insurance medical experts (*N* = 21), those who agreed more with the new legal precedent tended to agree more that AUD was a disease (*r*_*s*_ = 0.39, *p* = 0.077) and ascribed higher off-set responsibility (*r*_*s*_ = 0.54, *p* = 0.012). All other correlations did not reach statistical significance.

For addiction-specialist therapists (*N* = 30), those who agreed more with the new legal precedent also agreed more that AUD was a disease like cancer (*r*_*s*_ = 0.55, *p* = 0.002), agreed less that AUD was due to a weak will (*r*_*s*_ = 0.46, *p* = 0.011) and tended to agree less that AUD was due to a weak character (*r*_*s*_ = 0.33, *p* = 0.075). Finally, they tended to ascribe lower on-set responsibility (*r*_*s*_ = 0.31, *p* = 0.094). All other correlations were not statistically significant.

## Discussion

The present research aimed to shed light on different stakeholder groups’ views of AUD in general and specifically in relation to disability insurance and the new legal precedent, wherein AUD is now considered an illness and may entitle affected individuals to a disability pension.

### Regard for AUD

Addiction-specialist therapists had higher regard for people with AUD compared to insurance medical experts and lawyers, with the finding corresponding to a large effect size. In other words, therapists found these patients to be more “enjoyable, treatable, and worthy of medical resources” ([37], p. 257). The latter two groups’ scores were very similar to the MCRS scores reported by Gilchrist and colleagues [43] for general psychiatry, whereas for the addiction-specialist therapists scores were higher by a margin of about seven points in our study compared to the sample in Gilchrist et al. The higher scores in the therapist group may reflect a country-specific effect (Switzerland was not included in Gilchrist et al.) related to the national Swiss drug policy introduced in the 1990s, which was predicated more around harm-reduction approaches (e.g. heroin-assisted treatment, [44]). The therapists’ experience with their clients above and beyond mere abstinence-oriented treatment may have resulted in long-term and positive experiences with SUD patients. For instance, goal alignment is an important component of therapeutic alliance [45] and predictive of greater treatment retention [46], but is not always possible in abstinence-only treatment. This might explain therapists' higher regard scores. Alternatively, or perhaps additionally, it is conceivable that response rates were especially high amongst therapists who are particularly passionate about AUD patients.

### Views and opinions about AUD

We were interested in the extent to which participants endorsed a (medical) disease view and a moralist view of AUD. The two perspectives were assessed separately since they are not mutually exclusive [26]. Whilst all three groups strongly agreed that AUD was a disease, they concurred significantly less with the statement that AUD was a disease like cancer in that an individual is not at fault. Additional post-hoc analyses suggested that lower agreement with the latter statement may be caused by participants’ view that AUD was, at least to some extent, a self-inflicted disease. This perception of self-infliction has also been observed by other researchers in professional groups [29], medical students [47], and laypeople [6, 23]. Research suggests negligible changes in stigmatising public beliefs and behaviours towards AUD [6, 48, 49] over time, with affected individuals being blamed for their illness considerably more (85%) than e.g. individuals with AIDS (68%) or depression (18%) [23]. That said, past research found that medical professionals may also hold stigmatising views or support views that blame the individual [26, 29, 30].

Moreover, all three groups endorsed the view of AUD as a disease of the psyche more strongly than the brain disease model of addiction, with therapists tending to agree more with this perception than the other two groups. This corresponds with other findings, in which the brain disease model of addiction received limited support from treatment providers [26].

Statements that are linked to a moral view of AUD (and, thus, align with the old legal practice) received considerably less support than the disease view of AUD in all stakeholder groups, with the median ranging from 1 to 2 for the relevant items compared to a median of 5 for the statement that AUD was a disease. Support on all three items was higher amongst lawyers than addiction-specialist therapists, with scores amongst insurance medical experts falling between the two other groups. Similarly, other evidence suggests that the general public, i.e. medical laypeople, agreed more strongly that addiction was due to weakness, as compared to mental health and addiction specialists [30]. It is possible that the old legal practice, which had been in force for several decades, may hold a long-standing effect on lawyers’ views. Further, legal experts’ medical knowledge usually follows unstructured pathways and relies heavily on the internet [50]. Using these sources, rather than medical literature around addiction, may render it harder for legal experts to revise their opinion and align with current scientific consensus.

No statistically significant differences between the groups were observed regarding perceived on- and off-set responsibility, with the majority of participants (approximately 60% of the overall sample) fully or partially disagreeing with the two statements. This proportion is considerably higher than the respective 30% and 34% reported by Schomerus and colleagues (6, Supplemental Materials) in the German public [40]. The extent to which there actually are differences between the public and our stakeholder groups, or whether the differences are related to differences in wording between the studies or selection bias in our stakeholders, requires further research.

By trend, addiction-specialist therapists were more optimistic that AUD could be treated successfully than lawyers. Simultaneously, they were less optimistic than lawyers that withdrawal treatment would permanently improve a person’s ability to work. This is consistent with practical experience, especially in the case of severe and long-term addiction with repeated unsuccessful treatment attempts [15]. The three groups did not statistically significantly differ in the extent to which they felt that, in principle, withdrawal was reasonable for a person with AUD. That said, both lawyers and insurance medical experts believed more strongly that the intervention aim should always be abstinence, as compared to addiction-specialist therapists. Whilst most therapists disagreed with an insistence on abstinence, we observed a bimodal distribution in the other two groups. The lower support amongst therapists for abstinence is in line with such a measure not being unequivocally favoured by research evidence. This is applicable when compared to other treatment approaches that are based on harm-reduction [51, 52], such as controlled drinking, particularly if the latter is accompanied by specific psychotherapy [53]. Ultimately, the appropriateness of a specific measure requires a careful individual diagnostic assessment, including psychiatric and somatic comorbidities and previous successful and unsuccessful therapeutic measures [54].

### The new legal precedent

Roughly 1.5 years after the verdict, the new legal precedent was relatively well-known amongst the three groups, with percentages ranging from 80% for addiction-specialist therapists to 95% for insurance medical experts. Across the whole sample, the new legal precedent was perceived mostly positively, with 92% answering “yes” or “somewhat yes” when asked whether they thought it was right in principle that a person who cannot work because of AUD was eligible for disability benefits. We observed no statistically significant differences between the groups.

All three groups felt imposing abstinence as a therapeutic measure was less appropriate than a reduction in drinking quantity. These findings suggest that, across all groups, a harm-reduction approach was preferred over abstinence. It is possible that this finding is related to the specific situation in Switzerland, as previously discussed. Working with harm-reduction approaches has been found to result in more favourable views of such a method [55]. Thus, the political landscape could affect practitioners’ (and perhaps even society’s) openness towards a harm-reduction approach in treating addiction. Alternatively, it is conceivable that for most respondents there is a direct contradiction in recognising AUD as a disorder with pathological value and, simultaneously, insisting that affected individuals overcome their illness to mitigate damages. Addiction-specialist therapists also felt that imposing a reduction in drinking quantity was less appropriate than partaking in therapeutic sessions. This difference, albeit descriptively observable, failed to reach statistical significance in the other two groups. Addiction-specialist therapists seemed to diverge on reducing drinking quantity though, whereas no bimodal distributions were observed in the other two groups. These split views in therapists could be, for example, related to different experiences with this specific approach.

No statistically significant difference was observed between the groups regarding their support for therapeutic sessions as a treatment measure. It was fairly well accepted across the groups, with 80% of all participants indicating they felt it was fully or somewhat appropriate (compared to 58% for reduction in drinking quantity). Addiction-specialist therapists were significantly less favourable to requiring a reduction in drinking quantity than the other two groups. By trend, they were also less in favour of requiring abstinence than insurance medical experts and, at a descriptive level only, lawyers. Overall, imposing a request for abstinence received little support, with only 18% of respondents indicating that they felt this measure was fully or somewhat appropriate.

We also explored associations between attitudes consistent with the reasoning of the old legal practice and participants’ support for the new legal precedent. For lawyers, medium to strong correlations were observed for all but one of the variables of interest: lower agreement with the disease view of AUD, higher agreement with items related to a moralist view of AUD, and stronger ascription of on-set responsibility were all associated with lower support for the new legal precedent. This tentatively indicates that there may be an association between legal experts’ views on AUD and their support for the new legal precedent. However, more research is needed to validate these results.

For insurance medical experts, agreeing less that AUD was a disease and ascribing lower off-set responsibility were both related to lower support for the new legal precedent, albeit the former was only by trend. No other correlations were statistically significant. This may be related to the somewhat smaller sample size in that group and the associated lower power to detect effects if they exist. For addiction-specialist therapists, agreeing less that AUD was a disease like cancer, tending to agree more that AUD was due to weak character, and agreeing more that AUD was due to weak will were all associated with lower support for the new legal precedent. Moreover, ascribing higher on-set responsibility was associated by trend with lower support as well. Thus, for both medical professions in this study, there were also some links existed between being less inclined to consider AUD as a disease (like cancer), being more inclined to blame the individual, and the perception that, at least to some extent, AUD is self-induced.

Therefore, taking measures to ensure that these groups receive relevant and medically up-to-date information on AUD (and, more generally, SUDs) remains important, despite the overall low support for the moral model. Reducing the above-mentioned problematic views may result in sizeable changes for people with AUD, as high support of the new legal precedent by stakeholders is likely to facilitate appropriate access to disability insurance benefits. Previous research has reported a correlation between resource allocation decisions and, for example, the view that AUD was self-inflicted [23], although the exact relationship between structural and individual stigma requires further research [56, 57].

### Strengths and limitations

Our findings address gaps in the literature regarding views and attitudes of relevant Swiss stakeholders in the context of disability insurance for AUD. To our knowledge, it is the first study to include insurance medical experts and one of the first to include lawyers. The observed dissimilarities between the different medical expert groups align with other research [28] and highlight the importance of differentiating between medical professions. A strength of our research is that it was conducted online, a factor that has previously been found to reduce social desirability when answering questions on sensitive topics [58]. Another strength is our recruitment across Switzerland and all three major language regions, which helps to ensure nationwide representativeness.

It did prove challenging, however, to encourage individuals to partake in the survey. It was particularly difficult to recruit GPs, which meant that we were unable to include this important stakeholder group in our analyses. The majority of GPs who participated either concurrently worked as addiction-specialist therapists or as insurance medical experts and were therefore allocated to one of these groups. Lower GP response rates compared to other professions have been reported elsewhere [30] and may, according to research and our own experiences, be related to lack of time and perceived limited personal relevance of the topic [59]. As GPs play an important gatekeeper role to more specialised treatment, renewed efforts to study this group should be undertaken.

The response rate was not very high and a response bias in the sample cannot be discounted. It is possible that our findings portray a more positive message than what might be found in a less self-selective sample, assuming that many of those choosing to participate were particularly interested in the subject. Although our findings could be at the more optimistic end of the spectrum, response rates were similar across groups, which strengthens our confidence in the between-group comparisons. In addition, the question arises whether seemingly contradictory responses (e.g. supporting the view that AUD is a disease whilst simultaneously agreeing less that it was a disease like cancer, or also showing some support for moral model views) indicate socially acceptable responding. Although we cannot discount this, such seemingly contradictory responses have been previously observed [26, 29, 30], and may simply be reflective of participants’ mixed personal views or views that are more nuanced than the questions can capture. The use of partially non-validated questions in this study should also be taken into account, although several questions were based on pre-existing questionnaires [30, 48] or used in previous studies for which acceptable or good reliability was reported [24, 39]. Moreover, the MCRS has shown good validity and reliability [37]. Nonetheless, our results should be confirmed by future research using validated questionnaires.

The sample size and violations of assumptions did not allow for complex modelling of the data and restricted both statistical power and the range of applicable statistical tests. Future studies are necessary with larger samples that could run multiple regressions and other, more complex analyses to further enhance our understanding of these stakeholder groups. Finally, whilst the findings are informative in a Swiss context, they may not be universally generalisable. Yet, the results provide interesting pointers for future research both inside and outside of Switzerland.

### Concluding remarks

In this investigation of the views and attitudes about AUD of three Swiss stakeholder groups important within the context of disability insurance, most differences were apparent between addiction-specialist therapists and lawyers, with insurance-medical experts often scoring between the two groups. Whilst moral views were endorsed considerably less than a disease view of AUD, there still seemed to be the conception that, to a certain extent, AUD was a self-inflicted condition. There was considerable support for the new legal precedent amongst all three stakeholder groups, and they all preferred harm-reduction oriented approaches over requesting complete abstinence as a measure to mitigate damages.

Perceiving AUD to be self-inflicted and holding other views in line with the moral model can result in lower resource allocation decisions [23] and be otherwise detrimental to the support of people with AUD [21, 22]. Our results support these notions; stakeholders’ views of AUD were associated, to differing extents in the individual groups, with opinions on the new legal precedent. This underlines the importance of making current medical information about AUDs and, more generally, SUDs readily available and demonstrates the potential practical relevance of stakeholders’ views.

Stigmatisation of SUDs has been found to affect various behaviours in health care providers in the past [9]. It is possible that, over time, the change in legal practice, which was also accompanied by a new jurisdictional understanding of addiction, will affect legal experts’ attitudes and views of AUD and better align them with the prevailing medical view. Nonetheless, it may be worthwhile to address this more proactively, for instance by providing legal experts with information on addiction geared towards this specific group in the form of leaflets or as a scientific publication in a law journal rather than a medical journal [50]. To maximise the effectiveness of an intervention to reduce stigmatisation of individuals with AUD, a contact-based approach could prove promising [35, 36]. However, the extent to which these views actually affect stakeholders’ behaviour in the context of disability insurance requires further investigation.

Other important questions for future research remain. First, could the Swiss national drug policy, which includes harm reduction approaches, promote acceptance of harm-reduction oriented measures to mitigate damages amongst the stakeholders? Alternatively, do stakeholders perceive an unresolvable conflict between affording a person disability benefits, but then asking them to try and overcome their illness as a mitigating measure? If the national drug policy could be identified as one of the reasons behind the attitudes observed in this study, this would highlight a further advantage of adopting such a policy and may act as a model for other countries. Second, future studies should investigate stakeholders’ views on the new legal precedent and the appropriateness of different measures to mitigate damages for other drugs, both (partially) legalised substances, such as cannabis, or illegal substances, such as heroin or cocaine. Moreover, it would also be beneficial to gain a deeper understanding of stakeholders’ views on other models (e.g. the psychological model) and their perception of the assessment of AUD/SUDs.

## Supplementary Information

**Additional file 1. Table S1.** Items on Views of and Opinions on AUD: German Original Wording and English Translation. **Table S2a.** Spearman Correlation Coefficients for Correlations Between Individual Variables and Attitude Towards the New Legal Precedent for Lawyers. **Table S2b.** Spearman Correlation Coefficients for Correlations Between Individual Variables and Attitude Towards the New Legal Precedent for Insurance Medical Experts. **Table S2c.** Spearman Correlation Coefficients for Correlations Between Individual Variables and Attitude Towards the New Legal Precedent for Addiction-Specialist Therapists.

## Data Availability

The dataset generated and analysed during the current study are available from the corresponding author (HW) on reasonable request.

## Abbreviations

AUD: Alcohol use disorder
GP: General practitioner
MCRS: Medical Condition Regard Scale
SUD: Substance use disorder
