# Supplementary material for: Alcohol use disorder and disability insurance in Switzerland: the attitudes and views of lawyers, insurance medical experts, and addiction-specialist therapists
Source: Subst Abuse Treat Prev Policy. 2022 Oct 27;17:69. doi: 10.1186/s13011-022-00495-x (PMC9615404; doi:10.1186/s13011-022-00495-x)
Supplement: Supplementary file 1 — Additional file 1. Table S1. Items on Views of and Opinions on AUD: German Original Wording and English Translation. Table S2a. Spearman Correlation Coefficients for Correlations Between Individual Variables and Attitude Towards the New Legal Precedent for Lawyers. Table S2b. Spearman Correlation Coefficients for Correlations Between Individual Variables and Attitude Towards the New Legal Precedent for Insurance Medical Experts. Table S2c. Spearman Correlation Coefficients for Correlations Between Individual Variables and Attitude Towards the New Legal Precedent for Addiction-Specialist Therapists. [file 13011_2022_495_MOESM1_ESM.docx]

# Alcohol use disorder and disability insurance in Switzerland: The attitudes and views of lawyers, insurance medical experts, and addiction-specialist therapists

Helen Wyler*^1^, Anja Maisch^1^, Thomas Berger^2^, Ueli Kieser^3^, Roman Schleifer^1^ and Michael Liebrenz^1^

^1^ Department of Forensic Psychiatry, University of Bern, Bern, Switzerland

^2^Department of Clinical Psychology and Psychotherapy, University of Bern, Bern, Switzerland

^3^Institute for Legal Studies and Legal Practice, University of St. Gallen, St. Gallen, Switzerland

# Author Note

*Correspondence concerning this article should be addressed to Helen Wyler, Department of Forensic Psychiatry, University of Bern, Falkenplatz 16/18, 3012 Bern, Switzerland. Email: [helen.wyler@unibe.ch](mailto:helen.wyler@unibe.ch)

**Supplemental Online Materials**

*Table S1. Items on Views of and Opinions on AUD: German Original Wording and English Translation*

| **No** | **German wording** | **English translation** |
| --- | --- | --- |
|  | **Disease view** | |
| 1 | Alkoholabhängigkeit ist eine Krankheit. | Alcohol dependence is a disease. |
| 2 | Alkoholabhängigkeit ist genauso eine Krankheit wie Krebs: Man kann nichts dafür, dass man sie hat. | Alcohol dependence is a disease just like cancer: you cannot help having it. |
| 3 | Alkoholabhängigkeit ist am besten als eine Erkrankung des Gehirns zu verstehen. | Alcohol dependence is best understood as a disease of the brain. |
| 4 | Alkoholabhängigkeit ist eine Erkrankung der Psyche. | Alcohol dependence is a disease of the psyche. |
|  | **Moral view** | |
| 5 | Eine Alkoholabhängigkeit ist Ausdruck von Charakterschwäche. | Alcohol dependence is an expression of weakness of character. |
| 6 | Eine Alkoholabhängigkeit ist Ausdruck von Willensschwäche. | Alcohol dependence is an expression of weakness of will. |
| 7 | Einer Person mit Alkoholabhängigkeit fehlt es an Selbstdisziplin. | A person with alcohol dependence lacks self-discipline. |
|  | **On- and offset responsibility** | |
| 8 | Eine Person mit einer Alkoholabhängigkeit ist selbst verantwortlich für die Entstehung ihrer Sucht. | A person with an alcohol dependence is responsible for the development of his/her addiction. |
| 9 | Eine Person mit Alkoholabhängigkeit ist selbst verantwortlich für die Bewältigung ihrer Sucht. | A person with alcohol dependence is responsible for managing their own addiction. |
|  | **Treatment-related beliefs** | |
| 10 | Eine Person mit einer Alkoholabhängigkeit kann erfolgreich behandelt werden. | A person with alcohol dependence can be treated successfully. |
| 11 | Ein Entzug ist für eine Person mit Alkoholabhängigkeit grundsätzlich zumutbar. | In principle, withdrawal is reasonable for a person with alcohol dependence. |
| 12 | Das Ziel einer Intervention bei einer Alkoholabhängigkeit sollte immer die Abstinenz sein. | The goal of an intervention for alcohol dependence should always be abstinence. |
| 13 | Wenn eine Person mit Alkoholabhängigkeit einen Entzug macht, führt dies zu einer dauerhaften Verbesserung der Arbeitsfähigkeit. | When a person with alcohol dependence goes through withdrawal, this leads to a permanent improvement of their ability to work. |

*Table S2a. Spearman Correlation Coefficients for Correlations Between Individual Variables and Attitude Towards the New Legal Precedent for Lawyers*

| Variable | 1 | 2 | 3 | 4 | 5 | 6 | 7 | 8 |
| --- | --- | --- | --- | --- | --- | --- | --- | --- |
| 1 agreement with new legal precedent | - |  |  |  |  |  |  |  |
| 2 is a disease | .442^*^ | - |  |  |  |  |  |  |
| 3 is a disease like cancer | .400^*^ | .144 | - |  |  |  |  |  |
| 4 weak will | -.751^***^ | -.282 | -.286^*^ | - |  |  |  |  |
| 5 weak character | -.451^*^ | -.197 | -.323 | .767^***^ | - |  |  |  |
| 6 lack of self-discipline | -.618^***^ | -.129 | -.616^***^ | .836^***^ | .695^***^ | - |  |  |
| 7 on-set responsibility | -.416^*^ | -.340 | -.573^**^ | .597^***^ | .559^**^ | .785^***^ | - |  |
| 8 off-set responsibility | -.042 | -.369 | -.288 | .123 | .241 | .213 | .433^*^ | - |

*Note.* ^*^ *p* < .05, ^**^ *p* < .01, ^***^ *p* < .001.

*Table S2b. Spearman Correlation Coefficients for Correlations Between Individual Variables and Attitude Towards the New Legal Precedent for Insurance Medical Experts*

| Variable | 1 | 2 | 3 | 4 | 5 | 6 | 7 | 8 |
| --- | --- | --- | --- | --- | --- | --- | --- | --- |
| 1 agreement with new legal precedent | - |  |  |  |  |  |  |  |
| 2 is a disease | .394 | - |  |  |  |  |  |  |
| 3 is a disease like cancer | .291 | .208 | - |  |  |  |  |  |
| 4 weak will | -.189 | -.010 | -.330 | - |  |  |  |  |
| 5 weak character | -.224 | -.122 | -.035 | .211 | - |  |  |  |
| 6 lack of self-discipline | -.016 | -.112 | -.484^*^ | .457^*^ | .258 | - |  |  |
| 7 on-set responsibility | -.122 | -.276 | -.273 | .205 | -.067 | .060 | - |  |
| 8 off-set responsibility | .530^*^ | .091 | .270 | .355 | .006 | .350 | .187 | - |

*Note.* ^*^ *p* < .05, ^**^ *p* < .01, ^***^ *p* < .001.

*Table S2c. Spearman Correlation Coefficients for Correlations Between Individual Variables and Attitude Towards the New Legal Precedent for Addiction-Specialist Therapists*

| Variable | 1 | 2 | 3 | 4 | 5 | 6 | 7 | 8 |
| --- | --- | --- | --- | --- | --- | --- | --- | --- |
| 1 agreement with new legal precedent | - |  |  |  |  |  |  |  |
| 2 is a disease | -.146 | - |  |  |  |  |  |  |
| 3 is a disease like cancer | .553^**^ | -.177 | - |  |  |  |  |  |
| 4 weak will | -.459^*^ | .119 | -.279 | - |  |  |  |  |
| 5 weak character | -.329 | .105 | -.329 | .657^***^ | - |  |  |  |
| 6 lack of self-discipline | -.272 | -.009 | -.391^*^ | .629^***^ | .597^***^ | - |  |  |
| 7 on-set responsibility | -.312 | .091 | -.536^**^ | .507^**^ | .507^**^ | .283 | - |  |
| 8 off-set responsibility | -.256 | -.207 | -.237 | .398^*^ | .460^*^ | .439^*^ | .460^*^ | - |

*Note.* ^*^ *p* < .05, ^**^ *p* < .01, ^***^ *p* < .001.
